# Changes in MLST profiles and biotypes of *Corynebacterium diphtheriae* isolates from the diphtheria outbreak period to the period of invasive infections caused by nontoxigenic strains in Poland (1950–2016)

**DOI:** 10.1186/s12879-018-3020-1

**Published:** 2018-03-09

**Authors:** Urszula Czajka, Aldona Wiatrzyk, Ewa Mosiej, Kamila Formińska, Aleksandra A. Zasada

**Affiliations:** Department of Vaccines and Sera Evaluation, National Institute of Public Health – National Institute of Hygiene, Chocimska 24, 00-791 Warsaw, Poland

**Keywords:** *Corynebacterium diphtheriae*, Diphtheria, Invasive infections, MLST

## Abstract

**Background:**

*Corynebacterium diphtheriae* is a re-emerging pathogen in Europe causing invasive infections in vaccinated persons and classical diphtheria in unvaccinated persons. In the presented study we analysed genetic changes in *C. diphtheriae* isolates collected in Poland from the period before the introduction of the mass anti-diphtheria vaccination to the present time when over 98% of the population is vaccinated.

**Methods:**

A total of 62 *C. diphtheriae* isolates collected in the 1950s–1960s, 1990s and 2000–2016 in Poland were investigated. Examined properties of the isolates included toxigenic status, presence of *tox* gene, biotype, MLST type (ST) and type of infection.

**Results:**

A total of 12 sequence types (STs) were identified among the analysed *C. diphtheriae* isolates. The highest variability of STs was observed among isolates from diphtheria and asymptomatic carriers collected in the XX century. Over 95% of isolates collected from invasive and wound infections in 2004–2016 belonged to ST8. Isolates from the XX century represented all four biotypes: *mitis, gravis, intermedius* and *belfanti*, but the *belfanti* biotype appeared only after the epidemic in the 1990s. All except three isolates from the XXI century represented the biotype *gravis*.

**Conclusions:**

During a diphtheria epidemic period, non-epidemic clones of *C. diphtheriae* might also disseminate and persist in a particular area after the epidemic. An increase of the anti-diphtheria antibody level in the population causes not only the elimination of toxigenic strains from the population but may also influence the reduction of diversity of *C. diphtheriae* isolates. MLST types do not reflect the virulence of isolates. Each ST can be represented by various virulent variants representing various pathogenic capacities, for example toxigenic non-invasive, nontoxigenic invasive and nontoxigenic non-invasive.

## Background

Before the introduction of the mass anti-diphtheria vaccination the bacterium *Corynebacterium diphtheriae* inspired fear and was called “the strangling angel” of children because it was a major killer of children occurring in epidemics of diphtheria that resulted in thousands of deaths. The name ‘strangling angel’ of children arose from the wing-shaped pseudomembranes that form in the oropharynx. Dislodgment and impaction of these pseudomembranes caused acute airway obstruction and sudden death [1]. During the course of the disease toxin-producing (toxigenic) *C. diphtheriae* locally colonise the mucosa and the produced toxin is absorbed into the bloodstream and distributed throughout the whole organism. It causes early damage to the fibres of the cardiac muscle and its inflammation, conduction disorders and, possibly, heart block as well as demyelination of nerves which leads to the paralysis of the palate and ocular muscles [2].

After the introduction of the common anti-diphtheria vaccination in Europe the number of diphtheria cases decreased extremely. But in the 1990s a big epidemic occurred in the Newly Independent States of the Former Soviet Union (NIS) which affected all European countries. During this epidemic most cases were recorded not in children but in adults [3]. Subsequently, at the end of the 1990s *C. diphtheriae* invasive infections started to emerge in Europe and America. The majority of them are bacteraemia and endocarditis caused by nontoxigenic strains with the mortality rate reaching over 40% [4–10].

In Poland the last case of diphtheria was recorded in 2000 and the first invasive infection caused by nontoxigenic *C. diphtheriae* was reported in 2004 [11]. From that time such infections are recorded every year [4].

In this study we analyse genetic changes in *C. diphtheriae* isolates collected in Poland from the 1950s – before the introduction of the mass anti-diphtheria vaccination – to 2016 when over 98% of the population is vaccinated. Results of the study might shed some light on the dissemination and evolution of *C. diphtheriae* in response to increasing anti-diphtheria immunity in the population after epidemic waves and after mass vaccination.

## Methods

### Bacterial isolates

A total of 62 *C. diphtheriae* isolates were investigated from the strain collection of the National Institute of Public Health – National Institute of Hygiene (Poland). Among them eight were isolated from respiratory diphtheria in the 1950s–1960s, three were isolated from respiratory diphtheria in the 1990s, six were isolated from currieries in the 1960s, 1997, 2000 and 2001, 28 were isolated from invasive infections (bacteraemia and endocarditis) in 2004–2016, 16 were isolated from local infections (wound infections) in 2004–2016 and one was isolated in 2015 with no data concerning the type of infection. The group of isolates from 2000 to 2016 includes all of the *C. diphtheriae* isolates collected in Poland from the beginning of 2000 to June 2016 (except one isolate from 2014 that is missing). There are no identified epidemiological links among cases of *C. diphtheriae* infection in this period. The isolates were collected in various parts of Poland (Table 1). The city where *C. diphtheriae* was isolated is not always a home city of the patient because collected clinical samples are usually sent to diagnostic centres located in bigger cities. Isolates from the 1950s–1990s are the only isolates from that period in the NIPH-NIH collection without any data concerning epidemiological links.

**Table 1:** *Corynebacterium diphtheriae* isolates in the presented study

Isolate ID	Biotype	Toxigenicity status	MLST type	Allelic profile	Year of isolation	City of isolation	Site of isolation or disease
78/E	*gravis*	–	ST8	3–5–6-5-3-3-6	2016	Kędzierzyn-Koźle	Blood
77/E	*gravis*	–	ST8	3–5–6-5-3-3-6	2016	Nowa Sól	Wound
76/E	*gravis*	–	ST8	3–5–6-5-3-3-6	2016	Łódź	Wound
75/E	*gravis*	–	ST8	3–5–6-5-3-3-6	2016	Bydgoszcz	Wound
74/E	*mitis*	–	ST407 (new)	3–5–3-6-30-3-2	2016	Nowa Sól	Wound
73/E	*gravis*	–	ST8	3–5–6-5-3-3-6	2016	Piotrków Trybunalski	Blood
72/E	*gravis*	–	ST8	3–5–6-5-3-3-6	2015	Ostrołęka	NK
71/E	*gravis*	–	ST8	3–5–6-5-3-3-6	2015	Warszawa	Wound
70/E	*gravis*	–	ST8	3–5–6-5-3-3-6	2015	Warszawa	Blood
69/E	*gravis*	–	ST8	3–5–6-5-3-3-6	2015	Olsztyn	Blood
68/E	*gravis*	–	ST8	3–5–6-5-3-3-6	2015	Toruń	Blood
67/E	*gravis*	–	ST8	3–5–6-5-3-3-6	2015	Nowa Sól	Wound
66/E	*gravis*	–	ST8	3–5–6-5-3-3-6	2015	Kielce	Blood
65/E	*gravis*	–	ST8	3–5–6-5-3-3-6	2015	Warszawa	Wound
54/E	*gravis*	–	ST8	3–5–6-5-3-3-6	2015	Warszawa	Wound
42/E	*gravis*	–	ST8	3–5–6-5-3-3-6	2014	Słupsk	Blood
41/E	*gravis*	–	ST8	3–5–6-5-3-3-6	2014	Warszawa	Blood
40/E	*gravis*	–	ST8	3–5–6-5-3-3-6	2014	Warszawa	Blood
39/E	gravis	–	ST8	3–5–6-5-3-3-6	2013	Gdynia	Wound
38/E	gravis	–	ST8	3–5–6-5-3-3-6	2013	Słupsk	Blood
37/E	gravis	–	ST8	3–5–6-5-3-3-6	2012	Poznań	Blood
36/E	gravis	–	ST8	3–5–6-5-3-3-6	2012	Warszawa	Wound
35/E	gravis	–	ST8	3–5–6-5-3-3-6	2012	Gdańsk	Blood
34/E	gravis	–	ST8	3–5–6-5-3-3-6	2011	Kraków	Blood
33/E	gravis	–	ST8	3–5–6-5-3-3-6	2011	Radom	Blood
32/E	gravis	–	ST8	3–5–6-5-3-3-6	2011	Sosnowiec	Blood
31/E	gravis	–	ST8	3–5–6-5-3-3-6	2010	Legnica	Blood
30/E	gravis	–	ST8	3–5–6-5-3-3-6	2010	Gdynia	Blood
29/E	gravis	–	ST8	3–5–6-5-3-3-6	2010	Gdynia	Wound
28/E	gravis	–	ST8	3–5–6-5-3-3-6	2010	Bydgoszcz	Blood
27/E	mitis	–	ST392 (new)	3–46–3-3-30-3-2	2010	Bydgoszcz	Wound
26/E	gravis	–	ST8	3–5–6-5-3-3-6	2009	Sosnowiec	Blood
25/E	gravis	–	ST8	3–5–6-5-3-3-6	2009	Kraków	Blood
24/E	gravis	–	ST8	3–5–6-5-3-3-6	2009	Gdynia	Blood
23/E	gravis	–	ST8	3–5–6-5-3-3-6	2008	Warszawa	Wound
21/E	gravis	–	ST8	3–5–6-5-3-3-6	2008	Gdynia	Blood
20/E	gravis	–	ST8	3–5–6-5-3-3-6	2008	Bydgoszcz	Blood
19/E	gravis	–	ST8	3–5–6-5-3-3-6	2008	Rzeszów	Blood
18/E	gravis	–	ST8	3–5–6-5-3-3-6	2007	Gdynia	Blood
17/E	gravis	–	ST8	3–5–6-5-3-3-6	2007	Bydgoszcz	Wound
16/E	gravis	–	ST8	3–5–6-5-3-3-6	2007	Warszawa	Wound
15/E	gravis	–	ST8	3–5–6-5-3-3-6	2007	Bydgoszcz	Wound
14/E	gravis	–	ST8	3–5–6-5-3-3-6	2007	Gdynia	Blood
13/E	gravis		ST8	3–5–6-5-3-3-6	2006	Bydgoszcz	Blood
12/E	*gravis*	–	ST8	3–5–6-5-3-3-6	2004	Warszawa	Blood
11/D	belfanti	–	ST65	6–7–10-12–9-12–15	2001	Suwałki	Carrier
10/C	belfanti	–	ST69	6–7–21-17-9-7-11	2000	Suwałki	Carrier
6/B	gravis	–	ST32	3–1–18-4-13-3-5	1997	Otmuchów	Carrier
7/B	mitis	+	ST44	14–2–23-1-2-14-2	1990s	NK	Diphtheria
8/B	intermedius	+	ST44	14–2–23-1-2-14-2	1990s	NK	Diphtheria
9/B	mitis	+	ST44	14–2–23-1-2-14-2	1990s	NK	Diphtheria
5/A	gravis	+	ST63	5–6–7-1-3-5-8	1960s	NK	Diphtheria
4/A	intermedius	+	ST155	4–2–2-1-2-2-2	1960s	NK	Diphtheria
1/A	mitis	–	ST26	8–2–16-1-3-3-12	1960s	NK	Carrier
2/A	mitis	–	ST26	8–2–16-1-3-3-12	1960s	NK	Carrier
3/A	mitis	–	ST26	8–2–16-1-3-3-12	1960s	NK	Carrier
62/S	*gravis*	+	ST25	5–6–7-6-6-3-8	1950s	NK	Diphtheria
59/S	*mitis*	+	ST25	5–6–7-6-6-3-8	1950s	NK	Diphtheria
58/S	*gravis*	+	ST25	5–6–7-6-6-3-8	1950s	NK	Diphtheria
57/S	*gravis*	+	ST25	5–6–7-6-6-3-8	1950s	NK	Diphtheria
56/S	*mitis*	+	ST393 (new)	8–3–5-2-1-4-4	1950s	NK	Diphtheria
55/S	*intermedius*	+	ST155	4–2–2-1-2-2-2	1950s	NK	Diphtheria

*NK* not known

### Biotyping and toxigenicity testing

The isolates were biotyped using the API Coryne test (BioMerieux, France) according to the manufacturer’s instruction. Toxigenicity was determined by conventional and modified Elek tests according to the WHO manual [12]. The presence of the diphtheria toxin gene was investigated by PCR as described previously [13].

### DNA extraction

DNA for all molecular tests was extracted using DNeazy Blood and Tissue Kit (Qiagen, Germany) according to the manufacturer’s instruction for gram-positive bacteria.

### Multilocus sequence typing

The Multilocus sequence typing (MLST) was conducted as described by Bolt et al. [14]. Seven housekeeping genes (*atpA*, *dnaE*, *dnaK*, *fusA*, *leuA*, *odhA* and *rpoB*) were amplified by PCR, and sequenced and compared with the sequences submitted to the MLST database website (http://pubmlst.org/cdiphtheriae/) to determine sequence types (STs). The clonal relationship between isolates under study was visualised by constructing the minimum spanning tree based on the categorical clustering of STs using BioNumerics version 6.6 software (Applied Maths, Belgium).

## Results

**Fig. 1:**
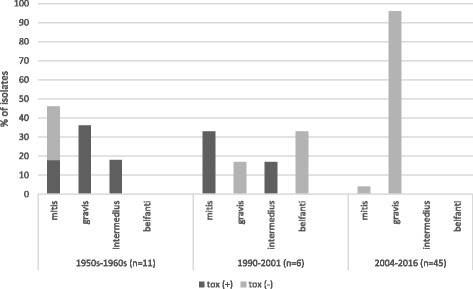
Distribution of *C. diphtheriae* biotypes in different time periods with consideration of toxigenicity of the isolates (n – number of isolates included in the study)

Among 62 *C. diphtheriae* isolates investigated 11 were toxigenic. The toxigenic isolates were collected in the 1950s, before the introduction of the common anti-diphtheria vaccination in Poland, and in the 1990s, during the epidemic in the former Soviet Union. Non-toxigenic toxin gene bearing isolates (NTTB) were not detected in the examined collection. The collection of isolates includes all biotypes: *mitis, gravis, belfanti* and *intermedius*. However, the majority of isolates belong to the biotype *gravis* – 48 of them. Nine isolates belong to the biotype *mitis*, three to *intermedius* and two to *belfanti*. Figure 1 presents the distribution of *C. diphtheriae* biotypes in time periods with consideration of the toxigenicity of the isolates.

A total of 12 sequence types (STs) were identified among the 62 *C. diphtheriae* analysed. Three of them: ST392, ST393 and ST407 were new types. The new types have been deposited in the *C. diphtheriae* MLST database (http://pubmlst.org/cdiphtheriae/). All three new types were represented by the *mitis* biotype and were isolated from a wound infection in 2010, respiratory diphtheria in the 1950s and a wound infection in 2016, respectively. Isolates from the 1950s–1960s represented ST25 (three *gravis* and one *mitis*), ST26 (three *mitis*), ST63 (one *gravis*), ST155 (two *intermedius*) and ST393 (one *mitis*) mentioned above. All isolates from this group except three *mitis* representing ST26 were toxigenic. Among four isolates from the 1990s three (two *mitis* and one *intermedius*) belonged to ST44 and were toxigenic, and one *gravis* belonged to ST32 and was nontoxigenic. Two nontoxigenic isolates from 2000 and 2001, representing the *belfanti* biotype, belonged to ST69 and ST65, respectively. All but three isolates from 2004 to 2016 belonged to ST8. All of these ST8 were biotype *gravis*. The three other isolates from this time period belonged to ST67 and, mentioned above, ST392 and ST407, and represented the biotype *mitis*.

**Fig. 2:**
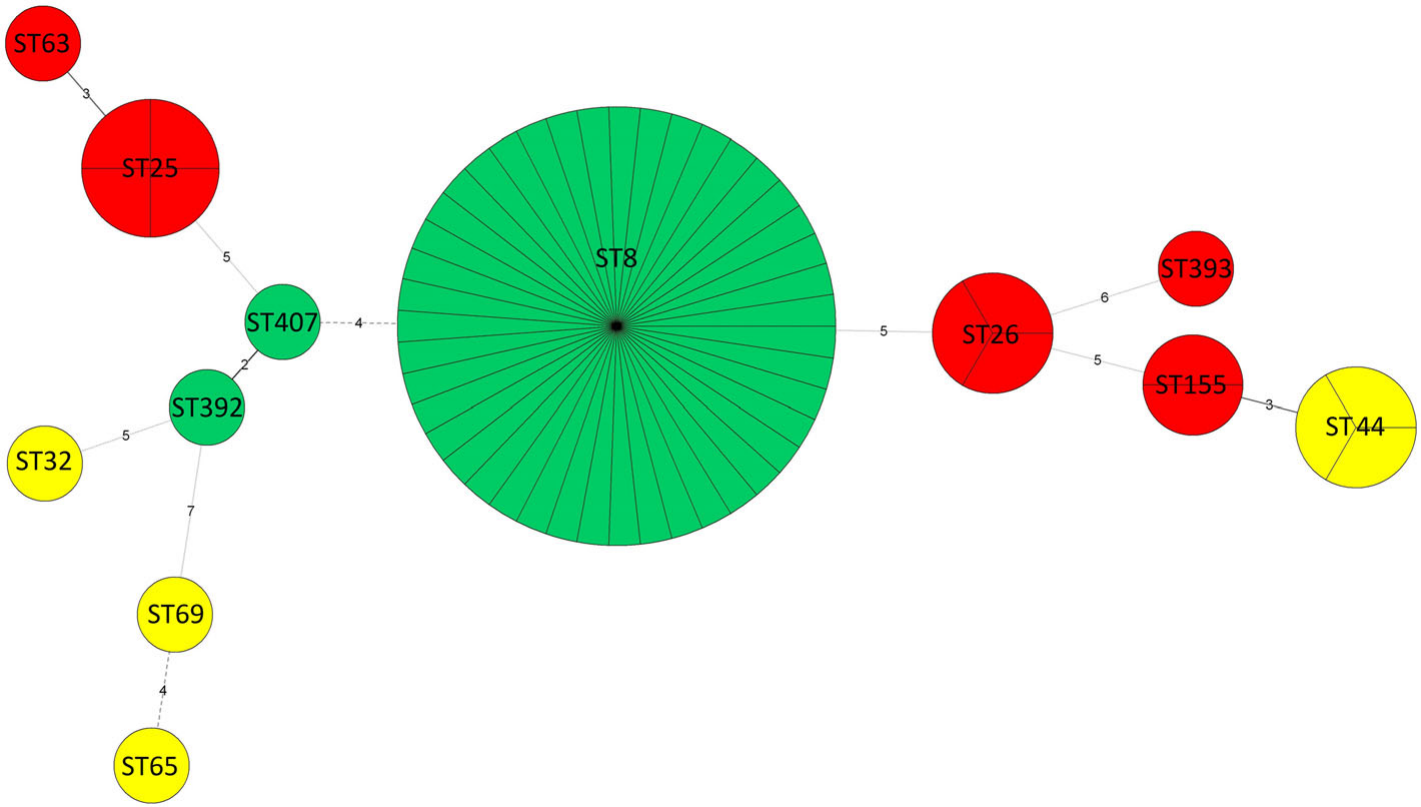
Minimum spanning tree of the MLSTs of the 62 *C. diphtheriae* isolates. Each circle corresponds to an ST. The area of each circle corresponds to the number of isolates. Each ST is colour coded according to its corresponding time period of isolation: red – 1950s-1960s, yellow – 1990s-2001, green – 2004-2016. The relationships between the isolates are indicated by the connections between the isolates and the lengths of the branches linking them. The numbers between the circles indicate the number of allelic differences

**Fig. 3:**
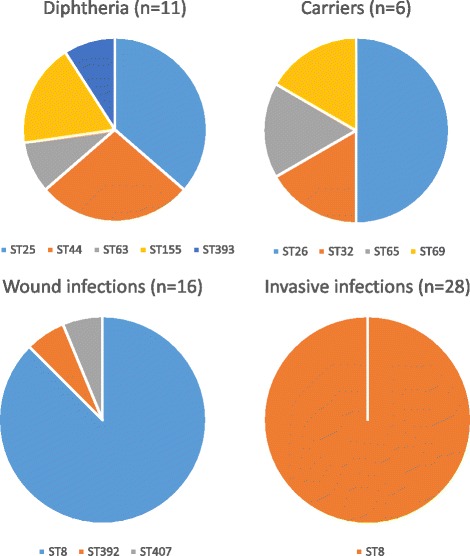
Distribution of STs by type of infections (n – number of isolates included in the study). The charts do not include isolate 72/E (site of isolation is not known)

Figure 2 presents a genetic correlation among the investigated isolates. The two closest related isolates were ST392 and ST407 isolated in 2010 and 2016, respectively, from wound infections, which share five of seven MLST loci. ST407 shares three loci with ST8 which represents the largest group of isolates collected in the same time period. Isolates from the 1990s-2001 and the 1950s–1960s showed significant genetic diversity. Figure 3 presents the distribution of STs by type of infections.

## Discussion

The number of diphtheria cases has been strongly limited in Europe due to the common anti-diphtheria vaccination. But the disease still exists in the world in endemic areas where diphtheria outbreaks occur. For example, according to World Health Organization data in 2015 diphtheria was recorded, among others places, in Madagascar – 1627 cases, The Lao People’s Democratic Republic – 194 cases, Myanmar – 87 cases, Haiti – 32 cases, Iran – 28 cases, Nepal – 26 cases, Thailand – 19 cases, but also in France – 14 cases and Germany – 14 cases [15]. The number of diphtheria cases in Europe increases every year [16]. The possible explanation for this is a decreasing level of the anti-diphtheria antibody with the increasing of age, the migration of people between Europe and diphtheria endemic countries and an increasing number of parents refusing to have their children vaccinated [16–21]. Moreover, life-threating invasive infections caused by nontoxigenic *C. diphtheriae* have appeared in Europe with increasing frequency [4, 7, 22–24]. All these facts point to the necessity of monitoring the spread and evolutional changes of *C. diphtheriae* in Europe.

When analysing the spread and evolution of *C. diphtheriae* in Poland it is worth keeping in mind the socio-demographical situation in this area, such as a limited number of refugees and immigrants, and not very strong anti-vaccination movements, yet. Due to high vaccination coverage there have been no diphtheria cases for over 15 years. *C. diphtheriae* strains circulating in Poland have been under strong selective pressure caused by the anti-diphtheria antibody level in the population. Moreover, a limited number of refugees and immigrants might be related to a limited number of strains imported from other regions of the world, including diphtheria endemic areas.

The presented MLST analysis of the *C. diphtheriae* collection revealed great genetic diversity among isolates from the 1950s–1960s. In this time period diphtheria was a common disease in Poland. In 1950–1956 there was a large diphtheria epidemic and, during its peak, the number of cases ranged from more than 24,000 to nearly 44,000 [25, 26]. The genetic diversity of toxigenic *C. diphtheriae* strains during an epidemic period is unusual. In other countries, like Algeria, the Russian Federation and other NIS, the Dominican Republic, and Haiti, epidemic waves were related to the domination of a particular genotype of *C. diphtheriae* [14, 27]. However, because of a limited number of isolates from that period we cannot exclude domination of a particular ST in Poland in 1950s–1960s. Due to the introduction of the mass anti-diphtheria vaccination in the whole area of Poland in 1954 the number of cases started decreasing rapidly. The increasing number of vaccinated persons in the population was related to replacing toxigenic strains with nontoxigenic as shown in Fig. 1. Interestingly, biotype *belfanti,* which is typically nontoxigenic, probably appeared in Poland after the epidemic in the 1990s. Similarly, in Algeria *C. diphtheriae* biotype *belfanti* was not isolated during the epidemic in 1992–1999. Then, in the post-epidemic period, the number of *belfanti* isolates started to increase reaching almost 100% of isolates in 2007–2015 [27].

In the 1990s there was a large epidemic in the former Soviet Union (FSU) which also affected other European countries. In Poland, several cases were recorded during this time. All toxigenic isolates from this time in the collection represented ST44. Interestingly, the clonal complex recognised as associated with the FSU epidemic composed of ST8, ST12, ST52 and ST66 isolates [14]. ST44 did not share any allele with the STs representing FSU epidemic clonal complex but it shared three alleles with ST155 toxigenic isolates collected in the 1950s–1960s in Poland. Borisova et al. [28] identified ST44 among eight STs of toxigenic *C. diphtheriae* isolated in Russia in 2002–2012. This might indicate that during the FSU epidemic diphtheria strains disseminated not only from the NIS to Europe but also from European countries, particularly Poland, to the NIS, where they have persisted till now. It is worth mentioning that after the fall of the Soviet Union in 1991 many people from Russia and other NIS travelled to Poland. It might enhance the transmission of C. diphtheriae strains between Poland and NIS.

On the other hand, ST8 isolates dominating in 2004–2016 in Poland and causing invasive infections also dominated in Russia in 2002–2012 together with two other STs causing diphtheria [28]. In both countries ST8 was represented by the *gravis* biotype, but in Poland the isolates were nontoxigenic. As ST8 was present in Russia for decades and it appeared in Poland 13 years ago, it might be hypothesised that the strain ST8 was transferred from Russia to Poland where it lost its ability to produce toxins as a result of environmental pressure of the vaccinated population (96%–99% of the Polish population received at least three doses of the anti-diphtheria vaccine [2]). Another possibility is an independent parallel evolution of *C. diphtheriae* strains in Poland and Russia. The latter hypothesis is supported by the fact that no NTTB isolates were identified in the examined collection. Mutations resulting in *tox* gene inactivation seem to be quite common in *C. diphtheriae* after epidemic waves [29] that can be an adaptation of the strains to the colonisation of the population with an increasing anti-diphtheria antibody level. We cannot exclude that NTTB strains were circulated in Poland before 2000 because of a limited number of investigated isolates from this period. But all isolates collected in Poland in 2000–2016 were included in the study which makes this group of isolates fully representative. We did not detect *tox* gene in the Polish ST8 isolates.

The ST8 nontoxigenic isolates are widespread in Poland [4]. Over 95% of *C. diphtheriae* isolates collected in various parts of Poland in 2004–2016 belong to this ST (Table 1). Moreover, all of the invasive infections were caused by ST8. However, isolation of one ST407 in 2016 and one ST392 in 2010 indicates that STs other than ST8 are also present in the Polish population, but their dissemination ability or pathogenic properties (or both) are significantly lower than ST8 isolates. Unfortunately, the status of the asymptomatic carriage of *C. diphtheriae* in the Polish population, that could provide information about non-pathogenic strains, is unknown because throat swabs are not routinely examined for this bacterium currently. Figure 3 presents the distribution of STs of isolates investigated in this study by type of infections. Despite a limited number of isolates collected in the period before the introduction of the anti-diphtheria vaccination, a great diversity of *C. diphtheriae* circulating in the population is visible. In that period at least three biotypes (*mitis, intermedius, gravis*) and various STs circulated, both toxigenic and non-toxigenic. Introduction of compulsory vaccinations against diphtheria caused not only the elimination of toxigenic strains but also reduced the diversity of *C. diphtheriae* isolates.

MLST is a valuable tool for the evolutionary investigation of bacteria as well as for tracking the spread of important clones. But this method offers limited usefulness for identification of hypervirulent clones. It is because MLST data are based on changes in the core genome whereas changes in virulence correspond primarily to changes in the accessory genome [30]. Sengal et al. [31] revealed that approximately one-third of the *C. diphtheriae* genome encodes accessory genes that vary widely between strains. The strains within one ST may differ in the presence of up to 290 genes. On the other hand, results of our study and the study presented by Borisova et al. [28] clearly suggest that *C. diphtheriae* ST8 may possess better adaptive properties and abilities to disseminate in current populations which seem to be independent from pathogenic properties. As the virulence factors determining invasive properties are not known, there is a possibility that ST8 strains circulating in Poland are represented by various virulent variants. We can assume that at least three virulence variants of ST8 circulate in Poland and Russia: toxigenic non-invasive (diphtheria), nontoxigenic invasive (bacteraemia, endocarditis) and nontoxigenic non-invasive (wound infection).

## Conclusions

The presented study has two main limitations: a relatively small number of isolates from the diphtheria epidemic periods (before the year 2000) and a lack of isolates from asymptomatic carriers after 2000. However, taking together the results of our study and results published by other researchers we can suggest that (i) nontoxigenic strains, particularly NTTB and the *belfanti* biotype, appear mainly after epidemic waves as a result of the increase of the anti-diphtheria antibody level in the population; (ii) during a diphtheria epidemic period non-epidemic clones of *C. diphtheriae* might also disseminate and persist on a particular area after the epidemic; (iii) increase of the anti-diphtheria antibody level in the population causes not only elimination of toxigenic strains but may also influence a reduction of diversity of *C. diphtheriae* isolates; (iv) each ST can be represented by various virulent variants. For these reasons it seems to be impossible to identify hypervirulent clones by MLST. Nevertheless, MLST might be useful in the identification of clones revealing better dissemination properties. To monitor effectively the spread of *C. diphtheriae* strains in Europe it is necessary to know STs circulating in each country. For further discrimination of STs, recognition of *C. diphtheriae* virulence determinants is necessary.

## Abbreviations

FSU: The Former Soviet Union
MLST: Multilocus Sequence Typing
NIS: Newly Independent States of the Former Soviet Union
NTTB: Non-toxigenic toxin gene bearing
ST: Sequence Type
